# Analysis of Embryoid Bodies Derived from Human Induced Pluripotent Stem Cells as a Means to Assess Pluripotency

**DOI:** 10.1155/2012/738910

**Published:** 2012-03-01

**Authors:** Steven D. Sheridan, Vasudha Surampudi, Raj R. Rao

**Affiliations:** ^1^Center for Human Genetic Research, Massachusetts General Hospital, Harvard Medical School, Boston, MA 02114, USA; ^2^Department of Chemical and Life Science Engineering, School of Engineering, Virginia Commonwealth University, Richmond, VA 23284, USA

## Abstract

Human induced pluripotent stem cells (hiPSCs) have core properties of unlimited self-renewal and differentiation potential and have emerged as exciting cell sources for applications in regenerative medicine, drug discovery, understanding of development, and disease etiology. Key among numerous criteria to assess pluripotency includes the *in vivo* teratoma assay that has been widely proposed as a standard functional assay to demonstrate the pluripotency of hiPSCs. Yet, the lack of reliability across methodologies, lack of definitive clinical significance, and associated expenses bring into question use of the teratoma assay as the “gold standard” for determining pluripotency. We propose use of the *in vitro* embryoid body (EB) assay as an important alternative to the teratoma assay. This paper summarizes the methodologies for creating EBs from hiPSCs and the subsequent analyses to assess pluripotency and proposes its use as a cost-effective, controlled, and reproducible approach that can easily be adopted to determine pluripotency of generated hiPSCs.

## 1. Introduction

 Human induced pluripotent stem cells (hiPSCs) are developing as exciting cell sources for applications in regenerative medicine [1] and drug discovery, primarily based on their extensive similarities to their human embryonic stem cell counterparts and shared properties of self-renewal and multilineage differentiation capabilities. The strategy of inducing pluripotency by activating the pluripotent network has proven to be successful in the reprogramming of somatic cells back to an embryonic-like state [2, 3]. However, there is a critical need to assess the pluripotent capabilities of the hiPSCs on a line-by-line basis once reprogramming has occurred to demonstrate this differentiation potential.

Multiple criteria have been proposed to evaluate the pluripotent state of generated hiPSCs [4–6]. Key among these criteria includes routine morphological analysis of cells for the presence of high nuclear-cytoplasmic ratio, cell surface and gene expression of pluripotent markers, demonstration of differentiation capabilities into derivatives from the three developmental germ layers (ectoderm, endoderm, and mesoderm), and specialized functional outcomes to demonstrate developmental potency. The functional assays developed thus far include *in vitro* differentiation, teratoma formation, chimera development, germline transmission, and tetraploid complementation [7]. The most stringent test to screen for pluripotency is the ability to demonstrate germline competency after chimera development, a test that can easily be conducted for mouse iPSCs. For hiPSCs, where testing for germline transmission and tetraploid complementation is not possible, teratoma formation of hiPSCs injected into immunocompromised mice and subsequent analysis of tissue formation has been widely used as an important methodology to investigate the developmental ability of the generated hiPSCs.

## 2. The Teratoma Assay for Pluripotency Assessment

Teratomas are solid, defined tumors, often germ-line derived, composed of the highly organized differentiated cells and tissues containing representatives of the three developmental germ layers that can also be generated artificially by transplanting pluripotent stem cells (hESCs or hiPSCs) into immunodeficient mice [8]. The histopathology of teratomas is remarkable in that they can serve as an important tool to observe early morphogenesis into organized tissues. The number of cells transplanted into the immunodeficient animal model in order to generate the fully differentiated teratoma *in vivo* can range anywhere between a hundred to a million cells or more [9]. As part of this methodology, the generated hiPSCs are usually transplanted at the following sites: intramuscular, subcutaneous, under the testis capsule, or under the kidney capsule in an immune deficient mouse. After a period of at least three weeks, the mature teratomas are excised out of the animal to be assessed for the presence of the cells derived from the three germ layers. The teratoma assay has been proposed to be the most stringent means to assess pluripotency of hiPSCs [10] but is not as rigid as the tetraploid complementation and germline transmission that can be conducted with mouse iPSCs [11]. In addition, the high costs associated with the assay, use of dozens or hundreds of animals, lack of a definitive clinical or biological relevance of the ability to form teratomas to the specific cell types subsequently derived from the hiPSCs, and the nonsystematic way this assay has been employed bring into question the use of the teratoma assay as the “gold standard” [4, 6, 8, 11].

Recent reports have highlighted the need to standardize the protocol to generate and examine the teratomas induced in the immunodeficient mouse models, if they are to be used as the gold standard for assessing pluripotency in generated hiPSCs [8, 10]. Studies have shown that some of the hiPSC lines have not been successful in forming all three germ layers in the teratomas but have been successful in deriving certain cell types that may have clinical significance [12]. Other researchers have also found that partially reprogrammed hiPSCs can form teratomas even if they do not meet other criteria for pluripotency, leading many to question the overall significance of the teratoma assay [6]. Generation of systematic protocols for teratoma generation and analysis that can be adopted across different labs also remains a challenge [8].

## 3. Embryoid Bodies as a Means to Assess Pluripotency of hiPSCs

An important alternative method to the teratoma assay is an *in vitro* approach involving the generation of embryoid bodies (EBs) from hiPSCs. EBs are three-dimensional aggregates of cells that are an amalgam of the three developmental germ layers [7]. In this approach, the undifferentiated hiPSCs are placed in suspension, which promotes stochastic differentiation into cells of all three germ layers. Formation of EBs is a routine approach used in the differentiation of the hiPSCs into different cell lineages [13]. One of the major advantages of this approach is that it is performed *in vitro* with standard tissue culture methods and materials, thus avoiding the regulatory issues and extensive expenses associated with maintaining immune-deficient mice. The EBs can be easily grown in a suspension culture in a petri dish in the laboratory and can be scaled up without much difficulty once the appropriate conditions for scale-up are established [14]. Unlike the teratoma assay in which hiPSCs that have passed all other pluripotency tests yet fail to form teratomas for unknown reasons [12], hiPSCs readily form EBs by a number of methods providing the ability to demonstrate trilineage differentiation and analysis in a more controlled, reproducible manner. The numerous approaches that have been developed to generate EBs along with the established analyses used for their pluripotent assessment are summarized in the following sections.

## 4. Methodologies for the Formation of hiPSC Embryoid Bodies

There have been several methods developed to create embryoid bodies for a variety of purposes, from the generation of specific tissue types to stochastic *in vitro* germ layer differentiation, to illustrate potency of candidate pluripotent stem cell lines [13, 15–17] (summarized in Table 1). For specific tissue lineages, EBs have been shown to be beneficial in the initiation of differentiation and to enhance the differentiation towards certain lineages [18] such as hematopoietic [15, 19], neural [20, 21], and cardiac tissues [22–25]. Methods for developing EBs differ in their ability to form aggregates of uniform size and the maintenance of their long-term viability. Typically, it is advantageous to control the uniform size of EBs for the reproducible differentiation of specific tissue types; however, the ability to form EBs of varied size for extended culture periods facilitates the formation of diverse tissues representing the three germ layers as a means to demonstrate differentiation potential. Both types of techniques can be used for the assessment of pluripotent stem line (hESC, hiPSC) quality.

### 4.1. Heterogeneous Methods of Embryoid Body Formation

**Figure 1:**
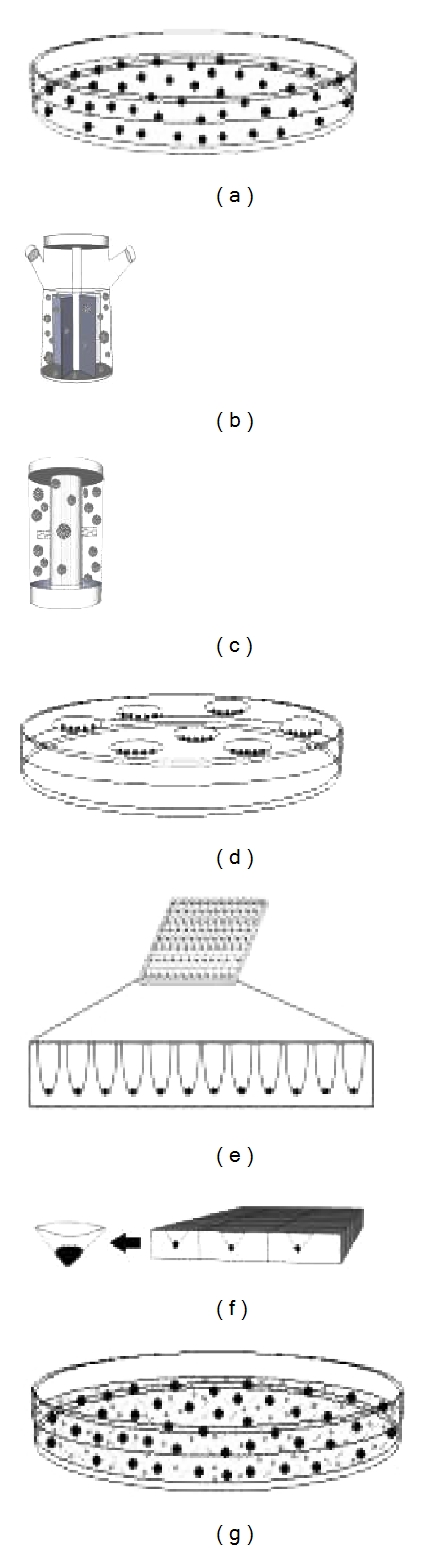
Schematic representation of methods to form embryoid bodies from pluripotent stem cells includes (a) liquid suspension culture, (b) stirred flask culture, (c) rotary cell culture systems, (d) hanging drop culture, (e) low adhesion U-bottom multiwall plates, (f) indented solid microsurfaces, and (g) hydrogel culture systems.

 Methods for the creation of stochastic EBs are the most straightforward and useful for the generation of varied germ layer representatives for subsequent demonstration of pluripotency [16]. Liquid suspension culture (LSC, [15]) is a common method for creating EBs and depends on the ability to grow cellular aggregates without attachment to the tissue culture vessel (Figure 1(a)). Typical tissue culture vessels for this purpose range from nontissue culture-treated petridishes to specially treated ultra-low attachment surfaces available through several vendors. These low-attachment tissue culture vessels are typically coated to provide a neutral, hydrophilic surface to prevent protein adsorption and subsequent cell attachment [26]. Hydrogels, such as naturally occurring agarose, or chemically defined, synthetic materials such as polyhydroxyethylmethacrylate (pHEMA) have been used to coat vessel surfaces to prevent the attachment of cells for the purpose of creating suspension cellular aggregates [26]. Though these surface treatments prevent the cell attachment to the tissue culture vessel resulting in aggregation, initial seeding densities need to be optimized in order to facilitate the cell-cell interactions required to form appropriately sized EBs. Seeding densities that are too low result in poor aggregation thus resulting in poorly established EB cultures. Thus, typical seeding densities are kept quite high during the initial aggregation (minimum of 10^6^ cells per 2 mL media in a 50 mm plate [16] or 6-well plate well). In the case of pluripotent hESC and hiPSC cultures, it is often desirable to facilitate the initial aggregation step by transferring isolated sections of colonies to low attachment vessels, since these cultures tend to be negatively effected by being in single cell suspension resulting in poor viability by apoptosis [35]. Once formed, suspension EBs cultures are typically cultured in the low attachment vessel for an extended period of time, typically from 28 to 35 days with fresh medium exchange every 2-3 days, though longer culture times may be desirable. Medium conditions have been demonstrated to have an important influence on the viability and germ layer differentiation of the suspended EBs. For instance, studies have shown that culture of EBs in a physiological glucose concentration (5.5 mM as opposed to 25 mM high glucose formulations typical of hESC and hiPSC expansion medium) in the presence of basic fibroblast growth factor (bFGF) prolonged the viability and increased the complexity of tissues in the EBs showing that representatives of all three germ layers in cultures could be maintained up to 105 days [36].

Though static suspension cultures of EBs are the most widely used due to their simplicity and the minimal cost of low-attachment treated standard vessels (dishes and multiwell plates), recent advances in bioreactor suspension culture have demonstrated usefulness of these techniques to optimize, standardize, and scale up the formation of EBs [17, 28, 29]. Unlike static suspension cultures, these systems require specialized culture equipment such as stirred flasks or the more recent Rotating Cell Culture Systems (RCCSs), developed by NASA, either the Slow Turning Lateral Vessel (STLV) or the larger capacity High Aspect Rotating Vessel (HARV) [37–39]. Typical stirred flask methodologies involve seeding hESC or hiPSC suspensions in a specialized flask utilizing a magnetic stirring bar to continually rotate the culture in order to facilitate aggregation of the cells into sustained cellular aggregates and provide better gas exchange than static systems [27] (Figure 1(b)). This method has been used to derive EBs and is amendable to scale up for larger yields [27]. Unlike these stirred flask suspension systems which allow for the EBs to be agitated over culture time allowing for greater gas exchange, less hypoxia, and less agglomeration than the static suspension system [27], the RCCS systems greatly reduce shear forces, present in the stirred flask systems, which can greatly damage the EBs [40, 41]. Often termed “microgravity” cell culture, RCCS systems allow for the continuous horizontal rotation resulting in very low shear stress on the cells, active membrane-based gas diffusion to prevent hypoxia and to equally distribute both oxygen and expiration waste gas, and can partially control EB size by regulating rotation speed and initial seeding densities (Figure 1(c)). Using these RCCS systems, the STLV in particular with its high membrane surface area to medium volume compared to the larger HARV, it has been demonstrated that EBs cultured in this fashion demonstrated higher viability, more complex tissue differentiation, and, in a specific example of neural induction, enhancement of neural progenitor differentiation [42]. 

### 4.2. Homogenous Methods of Embryoid Body Formation

 As opposed to the heterogeneous methods of EB formation that result in representatives of the three germ layers in a stochastic culture, it is frequently the case that more controlled EB formation methods for increased reproducibility and size control can facilitate the differentiation towards specific tissue types. In particular, control of EB size has been demonstrated to influence viability, proliferation, and differentiation potential [26] to cardiomyocytes [22, 25, 30, 43], endothelial tissue [43], as well as instruct hematopoiesis [19].

Several methods have been developed in order to form EBs of defined size. These methods share in their methodology ways to segregate a defined number of cells in order to allow them to aggregate before being collected for further culture. The hanging drop method utilizes 20–25 *μ*L drops containing a defined number of cells (typically 1000–10000) in single cell suspension [13, 30, 31]. The drops are placed onto the underside of a flipped 100 mm tissue culture plate lid, typically a maximum of 96 drops if using a multichannel pipette to place the drops (Figure 1(d)). Once placed, the lid is carefully flipped allowing the drops to remain attached to the lid and the lid is placed over a buffer-filled plate to prevent evaporation of the hanging drops. This technique is limited in the upper size limit of the initial cell aggregate due to the limited volume of the hanging drop required to allow fluid tension to adhere the drop to the lid (20 to 25 *μ*L). Initial formation of the cellular aggregates typically takes 1 to 2 days, after which the EBs are individually collected manually and placed in low-adhesion plates for further culture and maturation. A variation on the hanging drop using round bottom ultra-low attachment-treated multiwall plates has also been developed which allows for the derivation of larger EBs than the hanging drop method, in that larger amounts of cells can be placed into each well (i.e., 100–200 *μ*L of cell suspension per well in 96-well round-bottom plates) (Figure 1(e)) [14]. More recently, using a silicon wafer-based microfabrication technology containing hundreds or thousands of micrometer sized wells per cm^2^ adhered to the well bottoms of a standard multiwall plate, studies have demonstrated the ability to form large numbers of uniform and synchronized human EBs of defined size [32] in a commercially available format (AggreWell^TM^, Stem Cell Technologies) (Figure 1(f)). Though these techniques allow for the controlled aggregation of hESCs and hiPSCs, both require the formation of single cell suspensions exposing the cells to low viability and poor EB formation and, in the case of the plate based systems, centrifugation in order to force settle the cells into the bottom of the wells. It is common, therefore, that protective agents, such as the addition of Rho-associated kinase (ROCK) inhibitor, Y-27632, are required for these methods [35].

### 4.3. Other Embryoid Body Formation Methodologies

 The EB formation methods discussed previously represent the most commonly used for the purpose of assessing hESC and hiPSC pluripotency *in vitro*. Other more specific approaches exist for niche applications such as encapsulation techniques utilizing hydrogels such as methylcellulose [15, 33] or hyaluronic acid (HA) [34]. These techniques allow for the entrapment of single cells in suspension and subsequent growth of cellular aggregates (Figure 1(g)). These techniques have been mostly utilized for more specific goals than pluripotency assessment such as when cell clusters derived from single-cell clones are desirable. These techniques often suffer from very low yields [15] due to the intrinsic instability of prolonged single cell culture of the pluripotent stem cells as well as complicating the isolation of EBs from the hydrogel for subsequent downstream analyses.

## 5. Analysis of Germ Layer Formation in hiPSC-Derived Embryoid Bodies

 In order to assess the pluripotency of hiPSCs by the *in vitro* method of deriving EBs, it is imperative to have definitive downstream assays that demonstrate the ability to form representatives of the three developmental germ layers, thus demonstrating their increased differentiation potency upon reprogramming. There are several methods to show this that range from less stringent (showing expression of germ layer-specific genes) to the more stringent and definitive demonstration of tissue-similar structures (histology and immunohistochemistry) that resemble early embryonic development along with the concomitant expression of markers. This section will briefly review the typical requirements for such analyses.

### 5.1. Expression of Germ Layer-Specific Genes

 Perhaps the least stringent test of pluripotency in EBs is the demonstration of germ layer-specific gene expression by biochemical means. The reason for the lower stringency is that this only represents the ability to detect such gene expression without knowledge of higher-order structural or temporal expression within organized structures which can be discerned by more extensive histological and immunohistochemical examination (see the following). However, it is clear that the input cells, often fibroblasts in the case of their derivation toward hiPSCs, do not express these genes and can be used as an indicator of acquired tissue specific expression [61] (i.e., neural-specific genes being expressed). Gene expression analysis is also often used to demonstrate the lack of pluripotency genes upon EB differentiation, thus demonstrating the completeness of differentiation. This is of particular concern with hiPSCs, in order to demonstrate that the factors used to reprogram in the case of integrating retroviruses are in fact silenced [62]. There are several pluripotent and germ layer-specific markers used for the purpose of ascertaining differentiation within EBs (Table 2).

### 5.2. Histological Analysis of Embryoid Bodies

**Figure 2:**
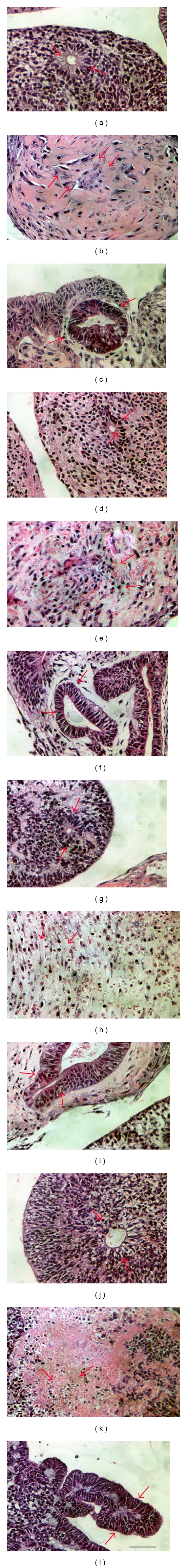
Histological evidence of germ layer differentiation in embryoid bodies generated from human pluripotent stem cells cultured by different methods to illustrate equivalence of culture techniques [63]. Shown are images of hematoxylin and eosin-stained histologic sections of EBs from hESCs propagated directly on mouse embryonic fibroblast (MEF) feeder layer (top row, a–c) or hESCs propagated in indirect coculture with MEFs (second row, d–f), hiPSCs propagated on MEFs (third row, g–i), or hiPSCs propagated in indirect coculture with MEFs (bottom row, j–l). Equivalent trilineage potential is demonstrated by presence of ectodermal (neuroepithelial) (a, d, g, and j); mesodermal (fibrous connective) (b, e, h, and k), and endodermal (intestinal) (c, f, i, and l) differentiation in these EBs. Arrows point to the corresponding tissue in each figure. Magnification is 400x total (10x ocular, 40x objective). Each scale bar represents 50 *μ*m in length.

**Figure 3:**
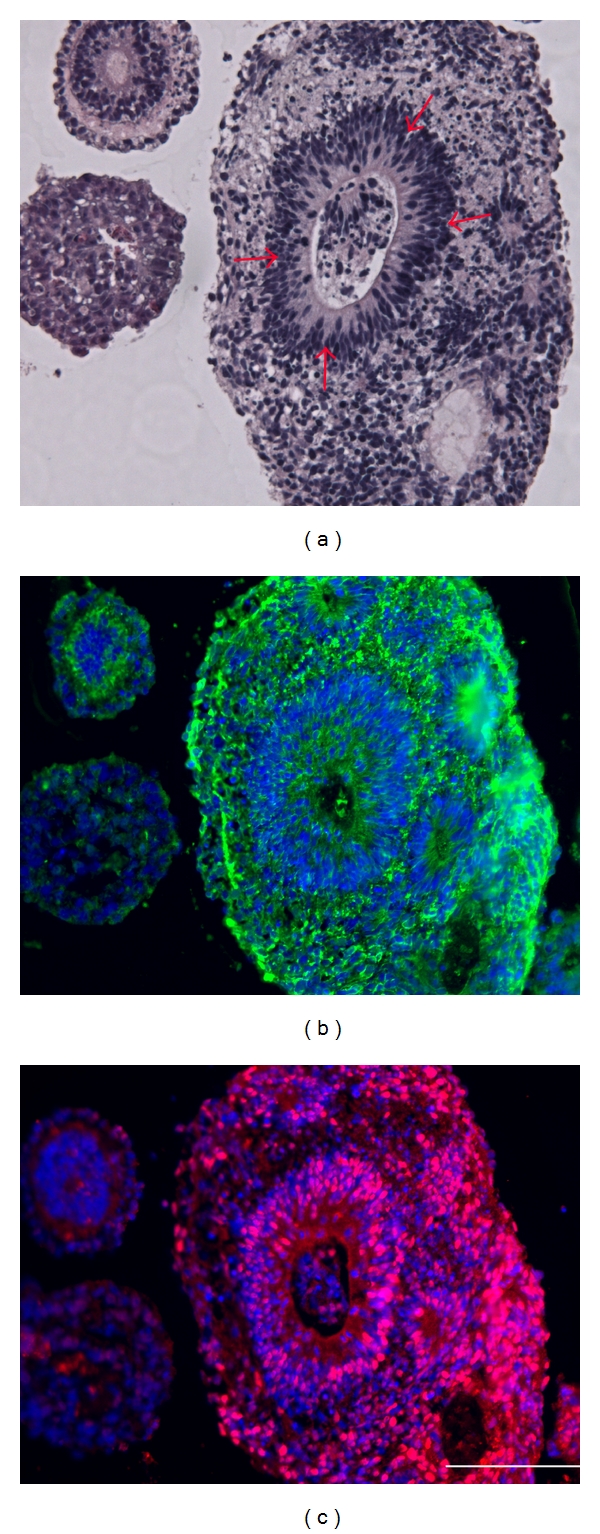
Immunohistochemical analysis of embryoid body sections confirming neuroepithelial tissue in adjacent sections, hematoxylin, and eosin-stained neural rosettes in hiPSCs-derived EBs (a), antinestin immunofluorescent staining (b), green-nestin, blue-nuclei stain, and anti-Sox2 immunofluorescent staining (c), red-Sox2, blue-nuclei stain. Magnification is 400x total (10x ocular, 40x objective). Each scale bar represents 50 *μ*m in length.

 Though gene expression analysis can be used to identify the presence of germ layer-specific markers and the concomitant lose of pluripotent markers, a more definitive assessment of tissue differentiation is based on the histological evaluation of sectioned EBs followed by assessment of tissue organization, cellular morphology, and localized protein expression [16, 28, 29, 36, 63]. Typically, EBs are first pelleted and embedded (i.e., low-melting point agarose) in order to concentrate the EBs for subsequent paraffin embedding and sectioning for mounting on microscope slides [63]. These slide-mounted sections are then stained with hematoxylin-eosin (H&E) in order to enhance contrast between the tissues and cells (Figure 2). Using these techniques, various tissues can be commonly recognized such as neural rosettes (ectoderm-Figures 2(a), 2(d), 2(g), and 2(j)), connective tissue (mesoderm-Figures 2(b), 2(e), 2(h), and 2(k)), and putative endoderm (Figures 2(c), 2(f), 2(i), and 2(l)). Though this technique is commonly used for both EBs and teratomas to determine the presence of germ layer representatives once their histomorphologies are identified, the assessment can be subjective and may require further immunohistochemical analysis in order to confirm the interpretation using specific antibodies (Table 2 and Figure 3) in sections adjacent to H&E stained sections. This is particularly true of putative endoderm, which is less prevalent and typically less mature in embryoid bodies than the more straightforward identification of neural and connective tissues. 

## 6. Conclusions

Although hiPSCs reprogrammed from human somatic cells have been well documented as a source of pluripotent stem cells with numerous shared similarities with hESCs, the increasing establishment of many new hiPSC lines requires the use of multiple assays and extensive resources to demonstrate their pluripotency on a line-by-line basis. Key among these is assays to demonstrate the potency towards formation of the three developmental germ layers and subsequent derivation of specific differentiated cell types in order to demonstrate their therapeutic potential. Recently, there has been enormous debate in the international stem cell community on the feasibility and use of the *in vivo *teratoma assay to demonstrate the pluripotency of derived hiPSCs [4, 6, 8–11]. Our paper proposes the embryoid body (EB) assay as a useful *in vitro*, cost-effective alternate to demonstrate the differentiation potential of derived hiPSCs. Methods for the generation of EBs and subsequent biochemical and histological analysis have gone through steady and tremendous improvements which permits its use across different laboratories. Further refinement and automation of the proposed methodologies provides opportunities for applications of the EB assay as a gold standard for assessing pluripotency of generated hiPSCs.

## Figures and Tables

**Table 1:** Common techniques to form human embryoid bodies.

	Controls EB size	Need for single cell suspension	Large-scale bioreactor production	Need for special equipment	References
Heterogeneous methods					
Liquid suspension culture	No	No	No	No	[15, 16]
Stirred flask culture	No	No	Yes	Yes	[27]
Rotary cell culture systems (RCCSS)	No	No	Yes	Yes	[28, 29]

Homogeneous methods					
Hanging drop culture	Yes	Yes	No	No	[13, 30, 31]
Low adhesion U-bottom multiwell plates	Yes	No	No	No	[14]
Indented solid microsurfaces (Aggrewell^TM^)	Yes	Yes	No	Yes	[32]

Other methods					
Hydrogels (e.g., methylcellulose, agarose, alginate)	No	Yes	No	No	[15, 33, 34]

**Table 2:** Typical markers for the analysis of human embryoid bodies.

	Marker name	Alternate name	Marker type	Suitable marker for gene expression	Suitable marker for IHC	Reference
Pluripotency						
	Oct4 (POU5F1)	Octamer-binding transcription factor 4	Transcription factor	Yes	Yes	[44]
	Nanog	n/a	Transcription factor	Yes	Yes	[45]
	REX-1	Zinc finger protein 42 homolog (*ZFP*42)	Transcription factor	Yes	Yes	[2]
	SOX-2	SRY (sex determining region Y)-box 2	Transcription factor	Yes	Yes	[46]
	SSEA-3	Stage-specific embryonic antigen 3 (SSEA-3)	Surface antigen	No	Yes	[47]
	SSEA-4	Stage-specific embryonic antigen 3 (SSEA-4)	Surface antigen	No	Yes	[47]
	Tra-I-60	n/a	Surface antigen	No	Yes	[48]
	Tra-I-81	n/a	Surface antigen	No	Yes	[48]

Ectoderm						
	GFAP	Glial fibrillary acidic protein	Glial intermediate filament	Yes	Yes	[49]
	Nestin	n/a	Intermediate filament	Yes	Yes	[49]
	Pax-6	Paired box gene 6	Transcription factor	Yes	Yes	[50]
	Sox-1	Sex determining region Y-box 1	Transcription factor	Yes	Yes	[51]

Endoderm						
	AFP	Alpha-fetoprotein	Plasma Protein	Yes	No	[52]
	Amylase	Alpha-amylase	Metabolic Enzyme	Yes	Yes	[53]
	FOXA2	Hepatocyte nuclear factor 3-beta (HNF-3B)	Transcription Factor	Yes	Yes	[54]
	PDX1	Pancreatic and duodenal homeobox 1, insulin promoter factor 1	Transcription factor	Yes	Yes	[55]
	GATA4	GATA binding protein 4	Transcription factor	Yes	Yes	[56]

Mesoderm						
	Brachyury	n/a	Transcription factor	Yes	Yes	[57]
	CD34	Cluster of differentiation molecule 34	Surface antigen	No	Yes	[58]
	FLT1	Vascular endothelial growth factor receptor 1	Surface receptor	Yes	Yes	[59]
	RUNX1	AML1, Runt-related transcription factor 1	Transcription factor	Yes	Yes	[60]
